# Heterotopic Pregnancy: A Difficult and Rarely Considered Diagnosis

**DOI:** 10.7759/cureus.36749

**Published:** 2023-03-27

**Authors:** Joseph M Crum, Howard W Levitin

**Affiliations:** 1 Emergency Medicine, OhioHealth Doctors Hospital, Columbus, USA

**Keywords:** ectopic, emergency medicine ultrasound, pelvic ultrasound, ovarian ectopic pregnancy, heterotopic pregnancy (hp)

## Abstract

A 34-year-old pregnant female presented to the emergency department (ED) with complaints of abdominal pain and vaginal bleeding for two days. The day prior, she was evaluated by her obstetrician and gynecologist with a transvaginal ultrasound demonstrating an intrauterine pregnancy at approximately six weeks gestation. After treatment of symptoms and reassuring laboratory testing, she went home. However, she returned two days later with worsening complaints. It was discovered that the patient had a heterotopic pregnancy, or a concomitant intrauterine and extrauterine pregnancy, resulting from natural conception in the absence of identifiable risk factors. While exceedingly rare, this diagnosis is frequently missed and associated with significant maternal morbidity and mortality if unrecognized.

## Introduction

Heterotopic pregnancy (HP), or the simultaneous presence of an intrauterine and ectopic pregnancy, is a rare and potentially life-threatening condition that requires a high index of clinical suspicion for timely diagnosis [1,2]. Although initially described in 1708 in an autopsy report, it was not until 1971 that it was first described in medical literature in the setting of increasing use of reproductive technologies [2,3]. These technologies, such as in vitro fertilization and ovulation induction therapy, have increased the incidence of HP to between 0.2% and 1% in medically assisted gestations. It remains exceedingly rare in natural conception cycles (<1/30,000 or 0.00003%) which contributes to its difficulty in establishing the diagnosis in low-risk populations [1]. Risk factors for HPs are the same as ectopic pregnancies and include a history of ectopic pregnancy, intrauterine device placement, or prior tubal ligation procedure [2]. Presenting symptoms range from being asymptomatic in 24% of cases to abdominal tenderness with hypovolemic shock in 13% of HPs [2,4]. While abdominal pain is the most common presenting complaint reported in the literature (72%), it carries an extensive differential that can further add to the difficulty in obtaining a timely diagnosis, which ultimately prolongs the initiation of definitive treatment.

## Case presentation

A 34-year-old female presented to the emergency department (ED) for evaluation of abdominal pain in the setting of first-trimester pregnancy from natural conception. She was a G3P2 at approximately six weeks gestation with an intrauterine pregnancy based on a transvaginal ultrasound performed by her obstetrician one day prior. Additionally, the ultrasound revealed a moderate amount of free fluid in the cul-de-sac which was deemed non-specific in the setting of an intrauterine pregnancy and no history of trauma. The patient’s pain persisted and increased in intensity prompting her trip to the ED. Recurrent emesis (non-bloody, non-bilious) and new-onset vaginal bleeding (not enough to soak through one sanitary pad) were reported.

Upon initial ED evaluation, the patient was mildly tachycardic with a heart rate of 105 beats per minute, but normal vital signs. Her abdomen was soft on examination with mild tenderness in her bilateral lower quadrants, without guarding or rebound. Laboratory testing demonstrated a hemoglobin of 11.7 g/dL and leukocytosis of 13.97 cells/µL. A urinalysis showed no evidence of infection or ketonuria. Her blood chemistry testing was normal (see Table 1). The patient was treated symptomatically (acetaminophen, metoclopramide, and diphenhydramine) and discharged home after the resolution of her symptoms. An outpatient follow-up was arranged after a discussion with her obstetrician and gynecologist (OB/Gyn).

**Table 1:** Laboratory workup on the initial ED visit. H: high; L: low; WBC: white blood cell; RBC: red blood cell; ED: emergency department

Lab	Value	Reference range
WBC count	13.97 (H)	4.5–11.00 K/µL
Absolute neutrophils	9.84 (H)	1.7–7.00 K/µL
RBC count	3.7 (L)	4.0–5.2 M/µL
Hemoglobin	11.7 (L)	12–16 g/dL
Hematocrit	34.3 (L)	36–46%
Mean corpuscular volume	92.7	80–100 fL
Platelet count	243	150–400 K/µL
Sodium	139	135–145 mmol//L
Potassium	3.7	3.5–5.1 mmol/L
Chloride	106	98–108 mmol/L
Bicarbonate	24	21–32 mmol/L
Anion gap	13	10–20 mmol/L
Glucose	91	65–99 mg/dL
Blood urea nitrogen (BUN)	8	8–25 mg/dL
Creatinine	0.54	0.40–1.10 mg/dL
Estimated glomerular filtration rate (eGFR)	124	>60 mL/minute/1.73 m^2^
BUN/Creatinine ratio	14.8	10.0–20.0
Calcium	8.9	8.4–10.2 mg/dL

**Figure 1:**
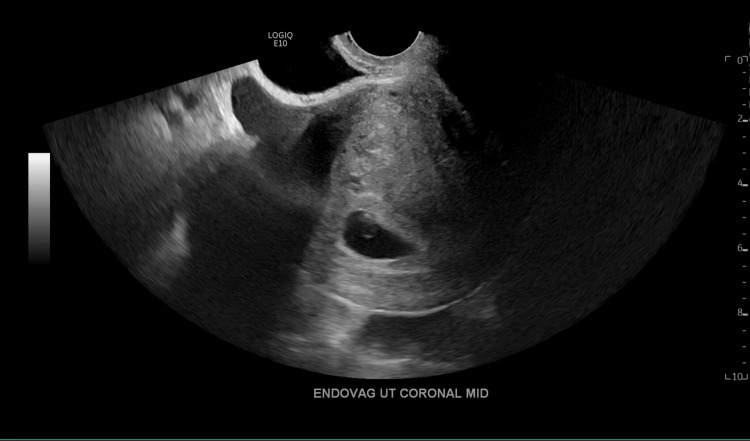
Transvaginal ultrasound in the mid-coronal plane demonstrating evidence of an intrauterine pregnancy with a visible gestational sac and yolk sac.

**Figure 2:**
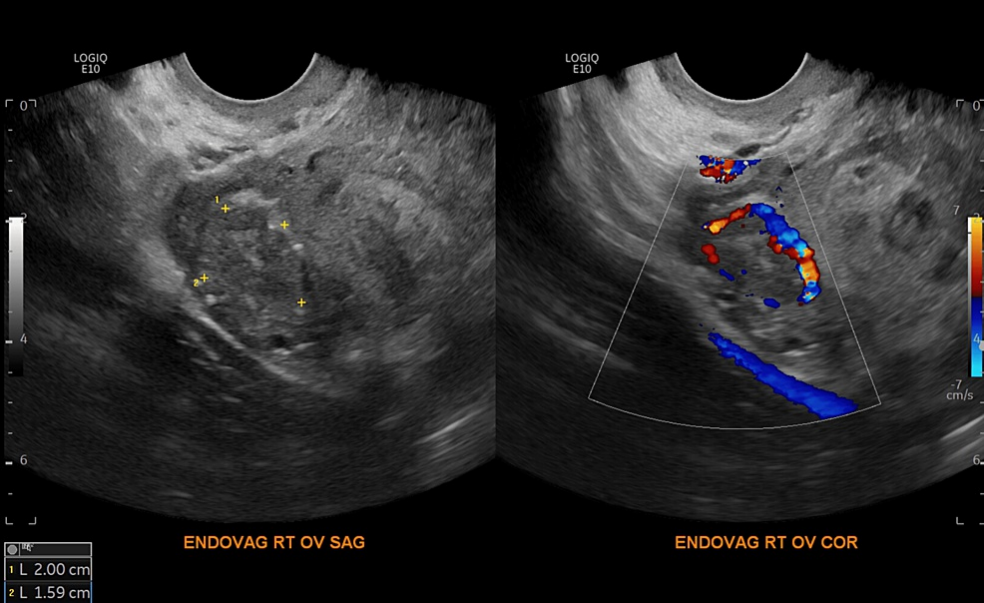
Transvaginal ultrasound of the right ovary in sagittal and coronal sections demonstrating a complex lesion with a “ring of fire” appearance using color Doppler.

Two days later, the patient returned to the ED with worsening abdominal discomfort on her right side, with an improvement in her nausea and vaginal bleeding. She was mildly tachycardic (pulse of 104 beats per minute) and hypertensive (151/92 mmHg) on initial evaluation, while her other vitals remained within normal limits. Physical examination was notable for significant tenderness to palpation in the right lower quadrant. Laboratory testing revealed an increasing leukocytosis to 15.94 cells/µL, although her hemoglobin was now within normal limits of 12.4 g/dL (see Table 2). Repeat transvaginal ultrasound reconfirmed an intrauterine pregnancy with a gestation of six weeks and one day, and demonstrated a new complex fluid collection within the pelvic cul-de-sac and a right adnexal lesion partially obscuring the right ovary (see Figures 1, 2). This new finding was thought to represent a recent corpus luteal cyst rupture.

**Table 2:** Laboratory workup on the second ED visit. H: high; L: low; WBC: white blood cell; RBC: red blood cell; ED: emergency department

Lab	Value	Reference range
WBC count	15.94 (H)	4.5–11.00 K/µL
Absolute neutrophils	12.51 (H)	1.7–7.00 K/µL
RBC count	3.82 (L)	4.0–5.2 M/µL
Hemoglobin	12.4	12–16 g/dL
Hematocrit	36.0	36–46%
Mean corpuscular volume	94.2	80–100 fL
Platelet count	289	150–400 K/µL
Sodium	138	135–145 mmol//L
Potassium	3.9	3.5–5.1 mmol/L
Chloride	104	98–108 mmol/L
Bicarbonate	20 (L)	21–32 mmol/L
Anion gap	18	10–20 mmol/L
Glucose	111 (H)	65–99 mg/dL
Blood urea nitrogen (BUN)	10	8–25 mg/dL
Creatinine	0.56	0.40–1.10 mg/dL
Estimated glomerular filtration rate (eGFR)	122	>60 mL/minute/1.73 m^2^
BUN/Creatinine ratio	17.9	10.0–20.0
Calcium	9.6	8.4–10.2 mg/dL

**Figure 3:**
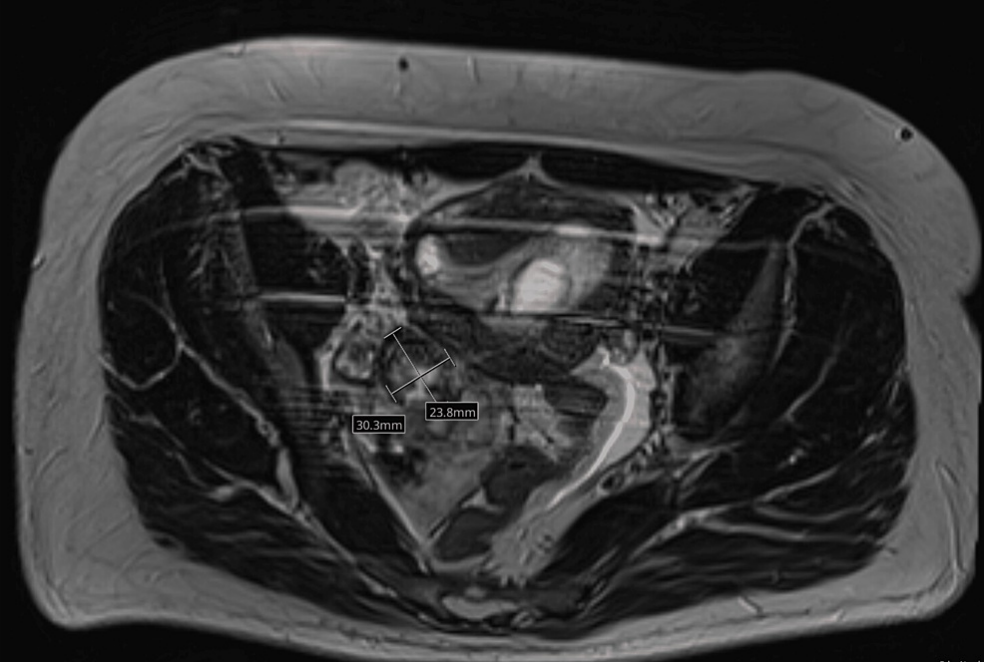
Cross-sectional MRI demonstrating a complex right adnexal lesion suggestive of an ectopic pregnancy with surrounding hemorrhage.

OB/Gyn admitted the patient for further evaluation and observation, while general surgery was consulted to rule out appendicitis. A joint decision by both services was made to obtain an abdominal MRI to better clarify the current differential for the patient’s progressive symptoms. This testing illustrated a right ovarian cystic lesion with morphology consistent with a gestational sac suggesting a “hidden” ectopic pregnancy (Figure 3). With these findings, the OB/Gyn service took the patient to the operating room for laparoscopic exploration. A heterotopic pregnancy was definitively identified within the right fallopian tube along with a moderate hemoperitoneum of approximately 300 mL of blood. The surgeon successfully removed her right fallopian tube, and the patient had an uneventful postoperative course.

The intrauterine pregnancy later proceeded to vaginal delivery at 36 weeks and three days gestation which was complicated by suspected placental abruption, pre-eclampsia, and postpartum hemorrhage requiring transfusion. The patient and child were ultimately discharged on postpartum day three.

## Discussion

This is a case of an HP resulting from natural conception in the absence of identifiable risk factors. While innovations in assisted reproduction technologies have increased the incidence of HPs, it remains a relatively rare and seldom considered diagnosis, particularly in a low-risk individual, as observed in this case. This patient had a reassuring initial ultrasound demonstrating a live intrauterine pregnancy at her outpatient OB/Gyn appointment the day before her initial ED visit, and three days before the definitive diagnosis. The only abnormality reported on this initial ultrasound was mild-to-moderate free fluid within the pelvis. While this finding is non-specific, it has been reported in isolated literature that having up to a moderate amount of free fluid within the pelvis correlates to a 52% chance of ectopic pregnancy [5]. This correlation increases to 86% if the fluid within the pelvis is quantified as moderate or greater. While it is generally believed that the presence of fluid in the pelvis during pregnancy is a common physiologic occurrence, this finding is associated with a 6.7% risk of pathology if there is no history of trauma [6].

While diagnostic laparoscopy with direct visualization remains the gold standard for identifying an ectopic pregnancy, transvaginal ultrasound is the preferred non-invasive alternative [7]. It is a sensitive and specific (84% and 98%, respectively) imaging modality that is widely available and lacks radiation risk to the mother and fetus.

MRI can be a useful adjunct in discerning abnormalities noted on the first-trimester ultrasound, particularly if there are equivocal findings. This diagnostic technology offers a high-definition evaluation of intra-abdominal and pelvic anatomy with increased soft tissue contrast and multiplanar imaging without the risk of ionizing radiation. The sensitivity of MRI ranges from 92% to 100% for the identification of ectopic pregnancy and is nearly 100% for detecting appendicitis [8]. Unfortunately, MRI still has limited availability in many locales, is costly, and requires considerable time to perform compared to ultrasound. In an unstable patient with a suspected ruptured ectopic pregnancy, emergent surgical intervention becomes the priority. In this case, the patient’s clinical and hemodynamic stability allowed time for investigation and MRI evaluation to appropriately identify the correct diagnosis. This approach further allowed the appropriate surgical team to intervene and remove the ectopic pregnancy.

## Conclusions

HPs remain an exceedingly rare and difficult diagnosis to establish. Closely monitoring the clinical picture, maintaining a broad differential diagnosis, and exploring abnormalities on first-trimester ultrasonography is key to identifying potential life-threatening pathology and instituting timely patient management. This is particularly true in the setting of pregnancy via reproductive-assisted technologies which are known to have a much higher incidence of HPs. MRI may also be beneficial in differentiating other causes of lateralizing abdominal pain in a stable pregnant patient.
